# *Leishmania* spp. seropositivity in Austrian soldiers returning from the Kosovo

**DOI:** 10.1007/s00508-019-01598-5

**Published:** 2020-01-07

**Authors:** Edwin Kniha, Julia Walochnik, Wolfgang Poeppl, Gerhard Mooseder, Adelheid G. Obwaller

**Affiliations:** 1 Institute of Specific Prophylaxis and Tropical Medicine, Center for Pathophysiology, Infectiology and Immunology, Medical University Vienna, Kinderspitalgasse 15, 1090 Vienna, Austria; 2 Department of Dermatology and Tropical Medicine, Military Medical Centre East, Austrian Armed Forces, Vienna, Austria; 3 Division of Science, Research and Development, Federal Ministry of Defence, Vienna, Austria

**Keywords:** Sand fly-borne pathogens, Leishmaniasis, Eastern Europe, Military, ELISA

## Abstract

Leishmaniasis is a severe vector-borne disease with two main clinical forms, visceral leishmaniasis and cutaneous leishmaniasis. Both forms of leishmaniasis are also endemic in Mediterranean countries including the Balkan region from where mainly visceral leishmaniasis is reported. Austrian soldiers returning from Kosovo were screened for anti-*Leishmania* antibodies to assess the risk of infection during operations. Anti-*Leishmania* antibodies were detected in more than 20% of the soldiers investigated, which indicates a considerable risk of infection during missions in this area and thus suggests the application of protective measures.

**Electronic supplementary material:**

The online version of this article (10.1007/s00508-019-01598-5) contains supplementary material, which is available to authorized users.

## Introduction

Leishmaniasis is a zoonotic disease complex, caused by various species of the protozoan genus *Leishmania* and transmitted by the bite of infected female sand flies. The visceral form (VL) of the disease is estimated to cause 300,000 infections and over 20,000 deaths every year. For the cutaneous form (CL), over one million cases were reported in the past 5 years [1].

*Leishmania infantum*, causing zoonotic visceral leishmaniasis and occasionally also cutaneous leishmaniasis, is widespread and re-emerging in the Balkan region [2]. Leishmaniasis is known to occur in all bordering countries of Kosovo, namely Albania, Montenegro, North Macedonia and Serbia, dogs being the main reservoir and maintaining the transmission cycle [2–6]. The Republic of Kosovo is disputed territory and approximately one third of the population live below the poverty line [7]. Despite leishmaniasis being notifiable in Kosovo, data on human and canine leishmaniasis are scarce and numbers from bordering countries suggest considerable underreporting [4]. Soldiers are generally at increased risk of infection for vector-borne diseases during missions in endemic areas. In a recent study, 8.6% of Austrian soldiers deployed in Bosnia and Herzegovina (BIH) showed antibodies against *Leishmania* spp. after returning [8]. In order to assess the exposure to *Leishmania* spp. and associated risk factors, soldiers of the Austrian Armed Forces (AAF) returning from the Kosovo were screened for anti-*Leishmania* antibodies.

## Methods

The Austrian Armed Forces (AAF) operate in Kosovo as part of the NATO mission Kosovo Force (KFOR). After return from a mission, soldiers are routinely subjected to a detailed health check. The AAF provided sera of 261 soldiers, taken immediately after their return from the Kosovo in 2013. All soldiers agreed to participate and to fill out a detailed questionnaire on demographic data (age, sex and place of residence), current and previous missions, duration of the missions, contact with animals in the mission area, insect bites, regular outdoor activities and holiday trips during the mission (see supplementary). The institutional review board of the AAF approved the study. All sera were tested for anti-*Leishmania* antibodies using the RIDASCREEN *Leishmania* AB enzyme-linked immunosorbent assay (ELISA) kit (R-Biopharm AG, Darmstadt, Germany), detecting IgM, IgG and IgA antibodies. The test was performed strictly following the manufacturer’s instructions. Results are shown as positive (index >1.1), borderline (index 0.9–1.1) and negative (index <0.9). Descriptive statistics were performed with a one sample proportions test and Fisher’s exact test. A two-sided *p* value <0.05 was considered statistically significant. Microsoft Excel 2011 for Mac (Microsoft, Washington, USA) and the R environment for Mac (R Foundation, Vienna, Austria) were used for data analysis.

## Results and conclusion

Of the 261 individuals included in this study, 258 (98.9%) were male and 3 (1.1%) were female. The collective comprised 240 (92.0%) professional soldiers, 20 (7.7%) militiamen and 1 (0.3%) civil servant. Participants were between 20 and 61 years old (median 28 years). Altogether, 55 out of 261 soldiers (21.1%, 95% confidence interval, CI 16.4–26.6%) had anti-*Leishmania* antibodies. Of these 36 (13.8%, 95% CI 10.0–18.7%) were positive and 19 (7.3%, 95% CI 4.6–11.3%) were borderline in the ELISA. These results clearly indicate that the soldiers are at risk of infection with *Leishmania* spp. during missions in Kosovo. Seropositivity did not significantly correlate with analyzed anamnestic data; however, seropositivity increased with the number of completed missions (Table 1). But also 28 of the 136 (20.6%, 95% CI 14.3–28.6%) soldiers for whom this was the first mission showed anti-*Leishmania* antibodies. Of these 17 (12.5%, 95% CI 7.7–19.5%) were positive and 11 (8.1%, 95% CI 4.3–14.4%) were borderline. Altogether, positive *Leishmania* ELISA indices ranged from 1.13 to 4.6. The results clearly demonstrate a considerable risk of exposure to *Leishmania *spp. in Kosovo, which is further corroborated by 1.7% out of 121 tested stray dogs from Kosovo showing anti-*Leishmania* antibodies in a recent study [4]. Military personnel are known to be a particularly vulnerable group for leishmaniasis because the soldiers are usually immunologically naïve compared to the local population in endemic areas and, spending most time outdoors in rural areas and sleeping in tents or simple housing, are highly exposed to sand flies. This has also been shown for Austrian soldiers returning from BIH, Syria and Lebanon [8].

**Table 1:** *Leishmania* seropositivity and number of prior missions

Prior missions	Positive (%)	OR/95% CI *p* value	Borderline (%)	OR/95% CI *p* value	Total ^a^ (%)	OR/95% CI *p* value
0	17/136 (12.5)	Reference	11/136 (8.1)	Reference	28/136 (20.6)	Reference
1–2	13/95 (13.7)	1.1/0.5–2.6 0.85	7/95 (7.4)	0.9/0.3–2.7 1	20/95 (21.1)	1.03/0.5–2.1 1
3–4	6/30 (20.0)	1.7/0.5–5.3 0.38	1/30 (3.3)	0.4/0.01–2.9 0.7	7/30 (23.3)	1.2/0.4–3.2 0.8
Total (%)	36/261 (13.8)	–	19/261 (7.3)	–	55/261 (21.1)	–

^a^ Positive or borderline

*OR* odds ratio, *CI* confidence interval

Of course, a positive ELISA index does not necessarily reflect a recent *Leishmania* infection. Anti-*Leishmania* antibodies and possibly also the parasites themselves, are known to persist for very long (occasionally life-long), even in asymptomatic infections [9–11]; however, in the current study, relatively high ELISA indices were observed. Moreover, the seroprevalence in soldiers returning from their first mission tripled the prevalence found in a large group of Austrian soldiers tested prior to missions [12]. Thus, although unfortunately, neither full blood samples for direct detection of *Leishmania* by PCR nor serum samples taken prior to the respective mission were available for proof, it seems to be justified to consider at least the high titers as infections acquired during this mission. Borderline results may be considered as infections acquired longer ago. Based on data from the Austrian normal population and data from previous studies on soldiers, most soldiers can be assumed to have travelled to endemic countries, particularly the Mediterranean region at least once before [8, 12]. None of the soldiers showed symptoms on return to Austria and also no significant association was observed between seropositivity and symptoms, e.g. fever and swollen lymph nodes, reported during the mission. Interestingly, in a comparable study by Obwaller et al. [8], although all soldiers reported being healthy, a positive association was found between seropositivity and a history of symptoms during the respective missions.

The awareness of the occurrence of the disease in Kosovo should be raised and protective measures, such as exposition prophylaxis and repellent usage, considered. Also asymptomatic infections may resurge in cases of immunosuppression, even after many years [13]. Moreover, Austrian soldiers are regular blood donors, usually twice a year. Several cases of blood transfusion-transmitted leishmaniasis (TTL) have been reported, especially in children [14]. In endemic countries such as Spain, high *Leishmania* seroprevalence rates among blood donors are reported and measures to prevent TTL, such as leukoreduction, have been implemented. Regular blood donation of military personnel with subclinical infections could pose a risk for an increased but unrecognized introduction of TTL infections, which particularly pose a risk for immunocompromized recipients of blood transfusions such as organ transplant recipients or cancer patients [15–17]. The U.S. Department of Defense and the American Association of Blood Banks (AABB) banned all U.S. soldiers who had served in Iraq from blood donation for 1 year [18].

Altogether, despite the abovementioned limitations and the limited specificity of the applied serological methods, the current study adds significant knowledge on the occurrence of and risk of infection with *Leishmania* in Kosovo and highlights the need of raising the awareness of the infection in general and for prophylactic measures in particular.

## Caption Electronic Supplementary Material

Questionnaire on demographic data filled out by military personnel participating in this study
