# Supplementary material for: Leishmania spp. seropositivity in Austrian soldiers returning from the Kosovo
Source: Wien Klin Wochenschr. 2020 Jan 7;132(1):47–9. doi: 10.1007/s00508-019-01598-5 (PMC6978428; doi:10.1007/s00508-019-01598-5)
Supplement: Supplementary file 1 — Questionnaire on demographic data filled out by military personnel participating in this study [file 508_2019_1598_MOESM1_ESM.pdf]

101

Fragebogen zur Studie Datum:_______________

1. Zur Person:

Geburtsjahr:_________ Geschlecht: M ☐ W ☐

Wohnsitz in Österreich: Postleitzahl (PLZ):_______________

Berufssoldat/in: ☐ Zivilbedienstete/r: ☐ Milizsoldat/in: ☐

Haben Sie beruflich Tierkontakt (z.B. Landwirtschaft, Jagd, Fleischverarbeitung, Küche, …) ☐ nein ☐ ja, wie?_________________________

2. Zum AuslE:

a) Aus welchem Einsatzgebiet kehren Sie zurück?______________________

b) Dauer des Einsatzes? _________________________

c) Waren Sie während des Einsatzes außerhalb des Einsatzgebietes auf Urlaub? wo?:______________________ wie lange?:___________________

d) Können Sie sich an <u>Mückenstiche</u> während des Aufenthaltes erinnern?

☐ nein ☐ wenige ☐ viele

e) Wie sind die Einstichstellen verheilt?

☐ rasch ☐ langwierig ☐ Besonderheiten:______________

f) Wurden Sie während des Einsatzes von einer <u>Zecke</u> gebissen?

☐ nein ☐ ja ☐ mehrmals

g) Hatten Sie während des Einsatzes Beschwerden wie:

☐ Fieber ☐ Kopfschmerzen ☐ Gliederschmerzen

☐ Husten ☐ Übelkeit/Erbrechen ☐ Erbrechen

☐ Sonstige: ___________________________________

h) Hatten Sie im Einsatz Kontakt zu Tieren? (z.B. Hunde, Mäuse, Ratten, ….)

☐ nein ☐ ja, welche?__________________________________

3. Allgemeine Fragen – unabhängig vom gerade beendeten AusIE:

i) Waren Sie schon vor diesem AusIE in anderen Einsatzgebieten des ÖBHs?

☐ nein ☐ ja, wo und wie lange?______________________________

j) Haben Sie Haustiere?

☐ nein ☐ ja, welche?____

k) Betreiben Sie regelmäßig Outdooraktivitäten? (z.B. Laufen, Wandern, Klettern, …)

☐ nein ☐ ja, welche?____

Vielen Dank für Ihre Teilnahme an der Studie!

Wenn Sie über das Ergebnis Ihrer Untersuchung persönlich informiert werden wollen, können Sie hier Ihre Kontaktdaten angeben:

Familienname: ____

Vorname: ____

Adresse: ____

Telefonnummer: ____

E-mail-Adresse: ____

Mit meiner Unterschrift bestätige ich, dass ich im Falle eines positiven Testergebnisses von der Studienleiterin persönlich informiert werden will:

Unterschrift des Teilnehmers: _________________________________
